# Quantitative analysis of interferon alpha receptor subunit 1 and suppressor of cytokine signaling 1 gene transcription in blood cells of patients with chronic hepatitis C

**DOI:** 10.1186/1743-422X-7-243

**Published:** 2010-09-18

**Authors:** Virginia Sedeño-Monge, Gerardo Santos-López, Rosa C Rocha-Gracia, Daniel Meléndez-Mena, Alberto Ramírez-Mata, Verónica Vallejo-Ruiz, Julio Reyes-Leyva

**Affiliations:** 1 Laboratorio de Biología Molecular y Virología, Centro de Investigación Biomédica de Oriente, Instituto Mexicano del Seguro Social, Km 4.5 carretera Atlixco-Metepec, CP 74360 Metepec, Puebla, México; 2 Posgrado en Microbiología, Centro de Investigaciones en Ciencias Microbiológicas, Benemérita Universidad Autónoma de Puebla, CP 72550 Puebla, Pue., México; 3 Servicio de Gastroenterología, UMAE, Hospital de Especialidades, Instituto Mexicano del Seguro Social, 2 norte 2004, CP 72000 Puebla, Pue., México; 4 Facultad de Medicina, Universidad Popular Autónoma del Estado de Puebla, 21 sur 1103, CP 72410 Puebla, Pue., México

## Abstract

**Background:**

Interferon (IFN)-α receptor 1 (*ifnar1*) and suppressor of cytokine signaling 1 (*socs1*) transcription levels were quantified in peripheral blood mononuclear cells (PBMC) of 59 patients infected with hepatitis C virus (HCV) and 17 non-infected individuals. Samples were obtained from patients infected with HCV that were either untreated or treated with IFN-α2 plus ribavirin for 1 year and divided into responders and non-responders based on viral load reduction 6 months after treatment. *Ifnar1 *and *socs1 *transcription was quantified by real-time RT-PCR, and the fold difference (2^-ΔΔCT^) with respect to *hprt *housekeeping gene was calculated.

**Results:**

*Ifnar1 *transcription increased significantly in HCV-infected patients either untreated (3.26 ± 0.31), responders (3.1 ± 0.23) and non-responders (2.18 ± 0.23) with respect to non-infected individuals (1 ± 0.34; *P *= 0.005). *Ifnar1 *transcription increased significantly (*P *= 0.003) in patients infected with HCV genotypes 1a (4.74 ± 0.25) and 1b (2.81 ± 0.25) but not in 1a1b (1.58 ± 0.21). No association was found of *Ifnar1 *transcription with disease progress, initial viral load or other clinical factors. With respect to *socs1 *transcription, values were similar for non-infected individuals (1 ± 0.28) and untreated patients (0.99 ± 0.41) but increased in responders (2.81 ± 0.17) and non-responder patients (1.67 ± 0.41). Difference between responder and non-responder patients was not statistically significant. *Socs1 *transcription increased in patients infected with HCV genotypes 1a and 1b (2.87 ± 0.45 and 2.22 ± 0.17, respectively) but not in 1a1b (1.28 ± 0.40). *Socs1 *transcript was absent in three patients infected with HCV genotype 1b. A weak correlation between *ifnar1 *and *socs1 *transcription was found, when Spearman's correlation coefficient was calculated.

**Conclusion:**

Our results suggest that HCV infection may up-regulate *ifnar1 *transcription. HCV genotypes differ in their capacity to affect *ifnar1 *and *socs1 *transcription, as well as in the ability to evade the antiviral response.

## Background

Hepatitis C virus (HCV) is a public health concern worldwide and a major cause of chronic liver inflammation, cirrhosis and hepatocellular carcinoma (HCC) [1]. In Mexico, the prevalence of HCV is ~1.4% in the open population and 35% in patients with active hepatitis [2].

HCV is a single-stranded positive RNA virus that codes for a precursor polyprotein, which is processed into 10 active proteins: C, P7, E1, E2, NS2, NS3, NS4A, NS4B, NS5A and NS5B. Due to high genetic diversity, HCV is classified according to several genotypes and subtypes, which differ in geographic distribution, virulence and sensitivity to medical treatment [3]. In Mexico, the prevalence of genotype 1 ranges from 30 to 87.5%, with a predominance of subtypes 1b and 1a. Genotypes 2 and 3 are less frequent and genotypes 4-6 are unusual in Mexican subjects [4,5].

Current therapy for HCV infection is the administration of pegylated IFN-α plus ribavirin for 24-48 weeks. However, almost 50% of treated patients do not respond to interferon therapy and, thus, are not able to clear the virus infection [3,6]. IFN-α activity is mediated by its high-affinity binding to IFN-α receptor (IFNAR) and subsequent induction of the Jak-Stat signaling pathway that activates transcription of >100 genes that establish an antiviral state in the cells [7].

The response to IFN-α therapy is influenced by HCV factors such as viral genotype, antigenic variability, viral susceptibility to IFN-induced proteins, expression of viral proteins that counteract IFN actions, etc. [8]. Indeed, HCV has developed several strategies to evade adaptive immune response and to block the action of effector proteins induced by IFN [9,10].

Some host genetic factors also affect the response to IFN-α therapy. In addition, the presence of anti-IFN-α antibodies and soluble forms of human IFNAR in plasma have been implicated in the resistance to IFN-α therapy in patients with chronic HCV infection [10-13]. Absence of or low intrahepatic transcription of *ifnar1 *is also related to a poor response to IFN-α and severity of liver disease [13-15]. Consequently, high expression of *ifnar1 *in liver and PBMCs of patients with HCV have been associated with efficient IFN-induced antiviral response and clearance of virus infection [16].

Virus infection induces the expression of negative regulators of the IFN signaling pathway such as the suppressor of cytokine signaling 1 (*socs1*), which associates with and inactivates Jak kinase, inhibiting the phosphorylation of both IFNAR and Stat proteins [17,18] and downregulating the transcription of IFN-stimulated genes [19]. Conversely, transfection of HCV core protein in mouse liver silences *socs1 *transcription leading to permanent activation of the Jak-Stat signaling pathway [20]. Transcriptional silencing of *socs1 *gene has been found in the liver of patients with chronic HCV infection and HCC [21].

Based on the significance of *ifnar1 *and *socs1 *genes in activation/downregulation of IFN-mediated antiviral response, we developed real-time RT-PCR assays to quantify *ifnar1 *and *socs1 *transcription in PBMCs of HCV-infected patients, testing their usefulness in the analysis of patients' response to IFN-α therapy.

## Results

Sixty-five HCV-infected patients were initially sampled. Samples of six patients were discarded because of their low leukocyte counts. The remaining patients were 35 females and 24 males with an age range of 20-69 years; 15/59 infected patients presented cirrhosis, 44 had active hepatitis, and none presented clinical data attributable to HCC.

Viral loads were monitored in all infected patients at 6 months after the end of treatment. Thirty-one infected and IFN-treated patients presented sustained viral response (responders, R) and cleared the virus infection and remained negative to viral genome detection. Seventeen IFN-treated patients did not reduce their viral loads (non-responders, NR). Eleven infected patients were analyzed prior to treatment and were considered untreated (U).

### Real-time RT-PCR assay validation

**Figure 1:**
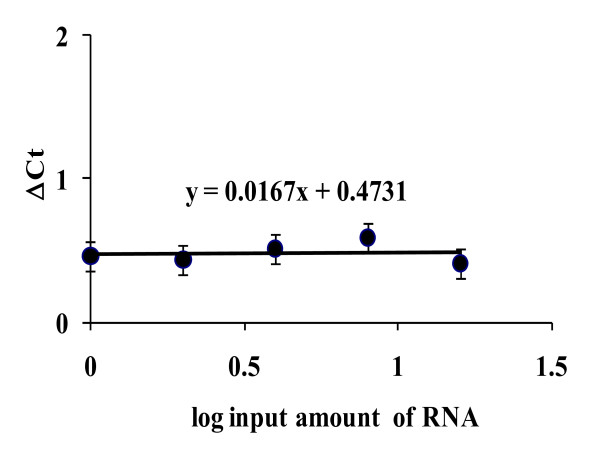
**Validation of *ifnar1 *real-time RT-PCR**. *Ifnar1 *detection assay was based on the use of TaqMan probes. Validation curves were performed with RNA extracted from HeLa cells, and the threshold cycle difference (**ΔCT**) between *ifnar1 *and the constitutive endogenous gene *hprt *was calculated and drawn with respect to RNA concentration. Slope value (**y**) indicates that *hprt *can be used for relative quantification of *ifnar1 *transcription.

A real-time RT-PCR assay was developed to quantify *ifnar1 *transcription based on the use of a TaqMan probe. To validate the detection method, we verified the amplification efficiencies of *ifnar1 *and the housekeeping gene *hprt *using RNA from human HeLa cell line. The differences of threshold cycle (ΔCT) between *ifnar1 *and *hprt *(CT_*ifnar1*_-CT_*hprt*_) at several mRNA concentrations gave a lineal slope of 0.0167 (Figure 1), indicating that both genes have proportional expression efficiencies within the dynamic range of 1-16 ng.

**Figure 2:**
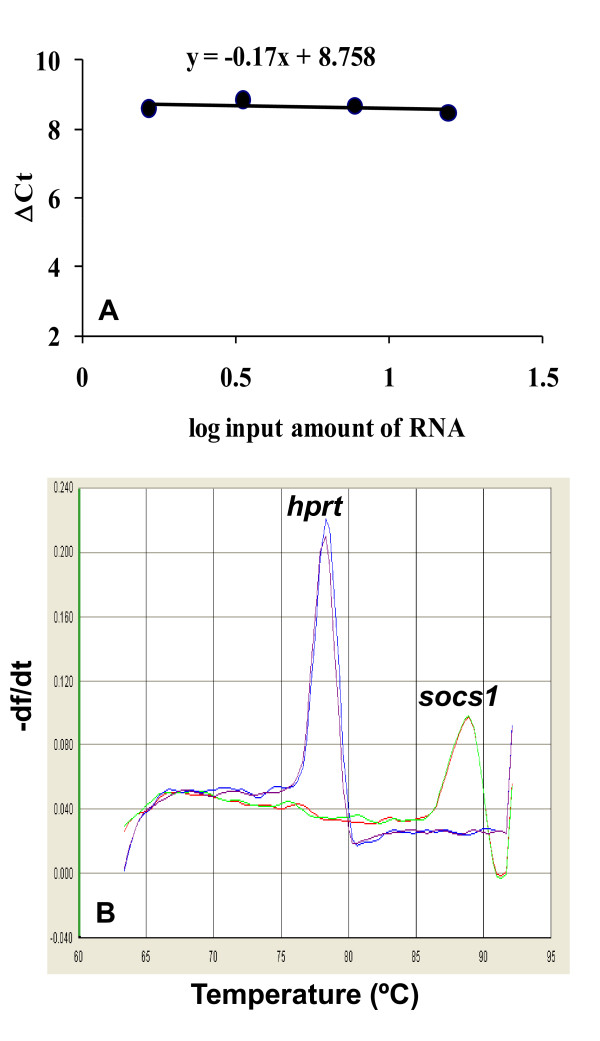
**Validation of *socs1 *real-time RT-PCR**. **A**. *Socs1 *detection assay was based on the use of Sybr Green. Validation curves were performed with RNA extracted from HeLa cells, and the threshold cycle (**ΔCT**) difference between *socs1 *and *hprt *was calculated and drawn with respect to RNA concentration. Slope value (**y**) indicates that *hprt *can be used for relative quantification of *socs1 *transcription. **B**. Assay specificity was corroborated by means of the dissociation curves. Peaks correspond to the melting temperatures for *socs1 *and *hprt *genes.

For the analysis of *socs1 *transcription, a quantitative method of real-time RT-PCR based on the use of SYBR Green was developed. Validation was performed by comparing the amplification efficiencies for *socs1 *and *hprt *genes in HeLa cells. Dynamic range for *socs1 *and *hprt *ΔCT values was 1.6-15.5 ng, giving a lineal slope of -0.17 (Figure 2A). Thus, *hprt *can be also used for the relative quantification of *socs1 *and permits to compare *socs1 *and *ifnar1 *transcription. The specificity of SYBR Green assay was corroborated by the dissociation curves that only showed the peaks corresponding to fusion temperatures for *socs1 *and *hprt *amplification products (Figure 2B).

### *Ifnar1 *transcription in HCV-infected patients

**Figure 3:**
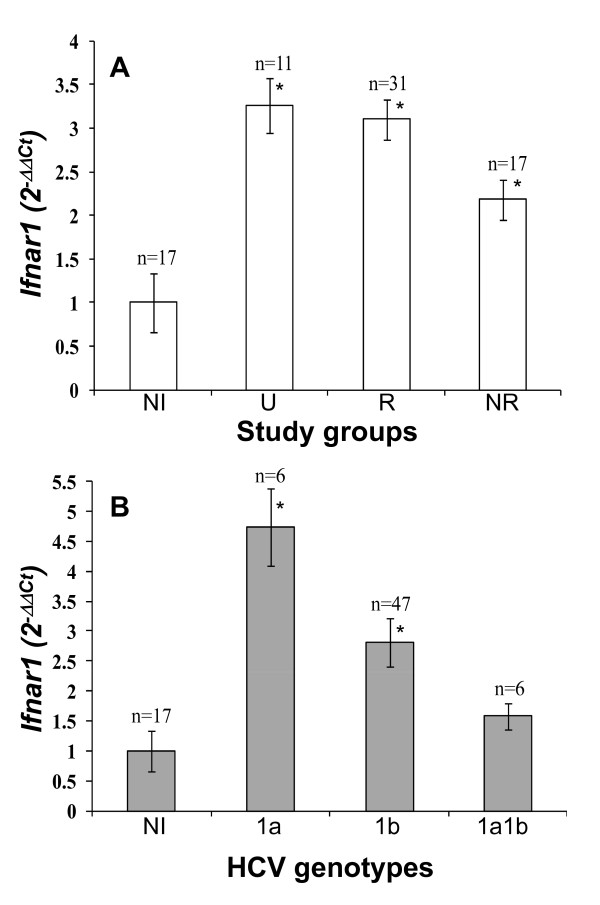
***Ifnar1 *relative transcription**. **A**. Relative quantification of *ifnar1 *transcription was determined by real-time RT-PCR and the fold difference (2^-ΔΔCt^) between study groups were calculated: non-infected controls (NI); untreated (U), responders (R) and non-responder (NR) patients. **B**. *ifnar1 *transcription in patients classified according to HCV genotype 1a, 1b and 1a1b (NI, non-infected).

For optimal relative quantification of *ifnar1 *mRNA the fold difference of ΔCT (2^-ΔΔCT ± SD^) between study groups were calculated. Resulting values were 1 ± 0.34 for uninfected individuals, 3.26 ± 0.31 for infected untreated patients, 3.1 ± 0.23 for infected IFN-treated responder patients and 2.18 ± 0.23 for non-responder patients (Figure 3A). These results indicate that *ifnar1 *transcription increased significantly (*P *= 0.005) due to HCV infection but was not significant with respect to the patients' viral response to IFN therapy.

We analyzed if viral load, ALT level or grade of liver disease was associated with *ifnar1 *transcription, but no correlation was found between *ifnar1 *transcription and any of these clinical factors (data not shown).

### Association of *ifnar1 *transcription and HCV genotypes

Infected patients were grouped according to their HCV genotype 1a (*n *= 6), 1b (*n *= 47) and 1a1b (*n *= 6). Other genotypes were not found. An association of *ifnar1 *transcription with viral genotype was found, thus the fold difference values for uninfected group were 1 ± 0.34, for genotype 1a 4.74 ± 0.25, for 1b 2.81 ± 0.25, and for 1a1b 1.58 ± 0.21 (Figure 3B). Increased transcription was statistically significant for genotypes 1a and 1b (*P *= 0.003) but not for 1a1b.

### *Socs1 *transcription in HCV-infected patients

**Figure 4:**
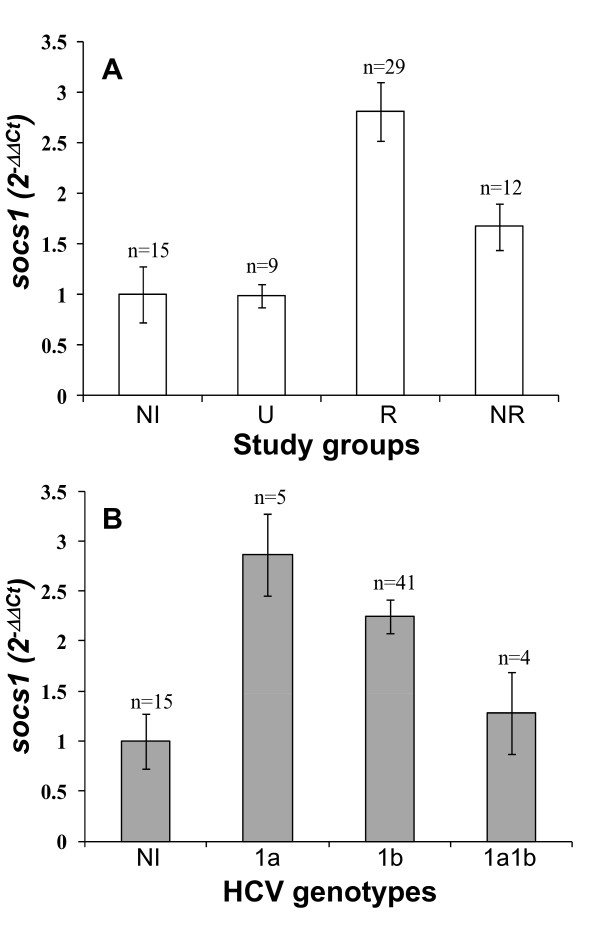
***Socs1 *relative transcription**. **A**. Relative quantification of *socs1 *transcription was determined by real-time RT-PCR and the fold differences (2^-ΔΔCt^) between study groups were calculated: non-infected controls (NI), untreated (U), responders (R) and non-responder (NR) patients. **B**. *Socs1 *transcription in patients classified according to HCV genotype 1a, 1b and 1a1b (NI, non-infected).

*Socs1 *transcription was analyzed in 15 non-infected individuals and 50 HCV-infected patients. These corresponded to nine untreated, 29 responders and 12 non-responder patients. The calculated fold differences between study groups were 1 ± 0.28 for non-infected individuals, 0.99 ± 0.41 for untreated subjects, 2.81 ± 0.17 for responders and 1.67 ± 0.41 for non-responders (Figure 4A). An apparent difference between responder patients and all other groups was noticed, but this was not statistically significant. No correlation was found between *socs1 *transcription and ALT levels, disease progression or viral loads before treatment.

### Association of *socs1 *transcription and HCV genotypes

For *socs1 *analysis, infected patients were grouped according to their HCV genotype 1a (*n *= 5), 1b (*n *= 41) and 1a1b (*n *= 4). *Socs1 *transcription increased in patients infected with HCV genotype 1a and 1b (2.87 ± 0.45 and 2.22 ± 0.17, respectively) but not in patients with genotype 1a1b (1.28 ± 0.41; Figure 4B). These differences were not statistically significant. S*ocs1 *transcripts were undetectable in 3/41 patients infected with HCV genotype 1b.

### Correlation between *ifnar1 *and *socs1 *gene transcription

**Figure 5:**
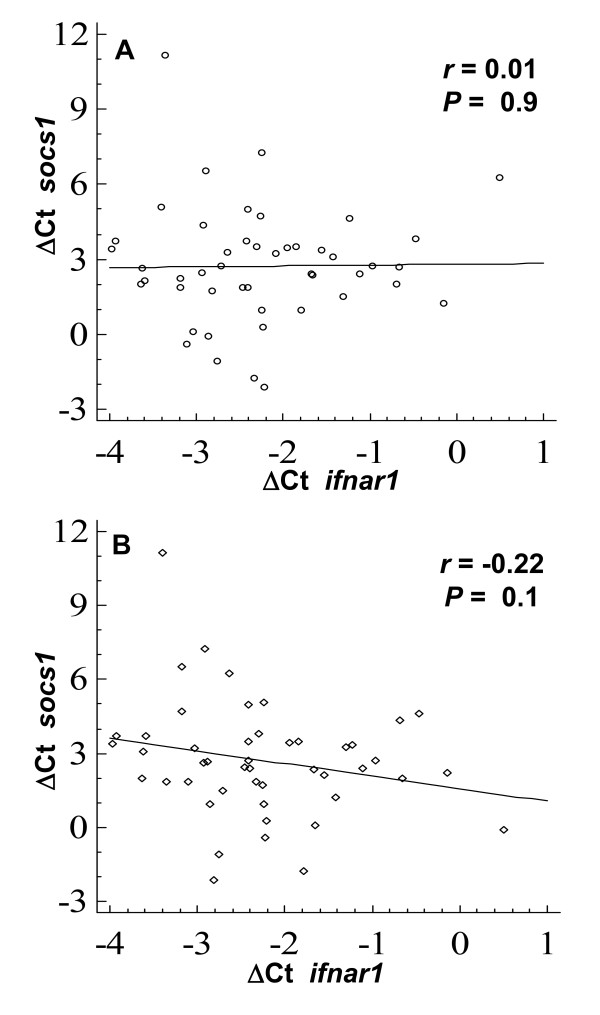
**Correlation between *ifnar1 *and *socs1 *gene transcription**. Spearman's correlation coefficient (***r***) between *ifnar1 *and *socs1 *ΔCT values was calculated. **A**. Analysis done with patients classified according with their response to treatment; non-infected controls (NI), untreated (U), responders (R) and non-responders (NR). **B**. Analysis done with patients grouped by HCV genotype.

The Spearman's correlation coefficient between *ifnar1 *and *socs1 *transcription was calculated. No correlation was found if patients were classified according with their response to treatment (*r *= 0.01, *P *= 0.9; Figure 5A). Instead, there was a weak inverse correlation between *ifnar1 *and *socs1 *transcription that was associated with virus genotype (*r *= -0.22, *P *= 0.1; Figure 5B).

## Discussion

We undertook this study to evaluate the association of *ifnar1 *and *socs1 *transcription with HCV infection and response to IFN therapy. We first validated the detection methods for both *ifnar1 *and *socs1 *transcripts and compared their amplification efficiencies with respect to the same housekeeping gene *hprt*. Based on their proportional expression within the dynamic range of 1-15 ng, we concluded that *hprt *can be used as a reference gene for relative quantification of *ifnar1 *and *socs1 *transcription as well as for comparative analysis of both genes, even though we used different strategies for their detection.

Results showed increased *ifnar1 *transcription in all groups of HCV-infected patients: 3.2- and 3.1-fold in the group of untreated and responder patients and only 2.1-fold in non-responder patients compared with non-infected individuals (*P *= 0.005). These results suggest that *ifnar1 *transcription increased due to HCV infection but was not significant for determining the association with response to treatment. We analyzed whether viral load, ALT level or grade of liver disease was associated with *ifnar1 *transcription, but no correlation was found between *ifnar1 *transcription and any of these clinical factors.

An association of *ifnar1 *transcription with viral genotype was found. Indeed, *ifnar1 *transcription was higher in infected patients than in non-infected individuals, 4.7-fold in patients with HCV genotype 1a, but only 2.8- and 1.6-fold in patients with genotypes 1b and 1a1b, respectively (*P *= 0.003). Viral genotype 1b is frequently associated with advanced liver disease and lack of viral response to IFN therapy; and the latter is further associated with reduced expression of IFNAR receptors, low functionality of the Jak-Stat pathway and reduced expression of proteins with antiviral activities. This is important because 14/15 patients with cirrhosis had HCV genotype 1b and 8/17 non responder patients had 1b genotype. Controversially, patients infected with HCV genotypes 1a1b had the lowest *ifnar1*-mRNA levels, but most of them responded to treatment. These results are not conclusive because of the scarce number of patients with genotypes 1a1b in this study.

Significant reduction of *ifnar1 *mRNA and protein levels in liver has been associated with high hepatitis activity, advanced liver fibrosis, poor response to IFN-α, and high viral load [15,22]. We and other authors carried out the quantification of *ifnar1 *in blood cells because it is less invasive and might be correlated with virus clearance in serum, mononuclear cells and probably the liver. Previous studies have analyzed the association of *ifnar1 *transcription in PBMCs with response to IFN therapy and reduction of serum viral loads using endpoint RT-PCR, but no correlation was found with viral clearance in liver [16,23]. Compared with those studies, our work improves *ifnar1 *quantification and sensitivity because of the use of real-time RT-PCR. However further experiments are required to correlate or not with the clearance of liver infection.

Experimental studies have shown that deficient production of *ifnar1 *transcripts may be one of the primary defects that lead to the lack of a response to IFN-α in HCV-infected patients [14,15]. Therefore, we also analyzed *socs1 *based on the hypothesis that *socs1 *contributes to the lack of response by down-regulating the Jak-Stat pathway and, consequently, *ifnar1 *transcription. At respect, *socs1 *gene promoter possesses transcription factor binding sites for STAT1, which both are activated by and initiate the negative feedback of the IFN-signaling pathway [24,25]. Our results suggested that the major inducer of *socs1 *transcription was exogenous IFN-α treatment but not virus infection as occurred with *ifnar1 *transcription. The levels of *socs1 *transcription were similar in non-infected individuals and infected patients who were not yet under IFN treatment, whereas in patients submitted to IFN-α treatment the level of *socs1 *transcription was higher. Although IFN therapy increased *socs1 *transcription, the effect was more notable in responders than in non-responder patients (Figure 4A). This is contrary to our initial hypothesis, but it is noteworthy that analysis of *socs1 *transcription can be used to pursue the response to treatment in other studies that include a higher number of patients.

We analyzed whether *ifnar1 *and *socs1 *transcription were associated, by calculating the Spearman's correlation coefficient. There was no association between *ifnar1 *and *socs1 *genes when patients were grouped on the basis of their response to treatment. However, these genes presented a weak inverse correlation, the lower *ifnar1 *the higher *socs1*, when patients were classified by viral genotype.

The role of *socs1 *in liver disease has been analyzed in mouse *socs1*^*-/+ *^submitted to chemically induced fibrosis, which in addition to *socs1 *reduction produces fatty degeneration of hepatic cells, macrophage infiltration and increases IL-6, TGF-β and IFN-β secretion. Those data indicate that liver reduction of *socs1 *increased with liver inflammation and fibrosis [26].

It is noteworthy that *socs1 *transcripts were absent in 3/41 patients infected with genotype 1b. Undetectable *socs1 *transcription is a frequent finding in liver biopsies of patients with HCC [21]. In addition, *socs1 *has been associated with tumor suppression because it reduces the expression of several genes involved in cell transformation [27-29] and silencing of *socs1 *gene leads to tumor progression [26]. In our study, none of the patients with undetectable *socs1 *transcripts presented cirrhosis or HCC; however, these results suggest that silencing of *socs1 *transcription started early during HCV infection.

## Conclusions

Our results suggest that HCV infection may up-regulate *ifnar1 *transcription. Viral genotypes differ in their capacity to affect *ifnar1 *and *socs1 *transcription; as well as in their ability to avoid the antiviral response. Further studies should be performed to test the role of HCV genotype 1b in the silencing of *socs1 *transcription.

## Methods

### Patients

Consecutive patients with chronic HCV infection (*n *= 59) were selected from the Gastroenterology Service of the High Specialty Medical Unit, Mexican Institute of Social Security in Puebla, Mexico. Blood samples were obtained from April 2008 to August 2009. Inclusion criteria were patients of both genders, age range 20-69 years, abnormal ALT levels, positive to anti-HCV antibodies and detectable HCV RNA in serum. Patients were clinically evaluated, and laboratory tests were performed to identify stage of liver disease. Patients coinfected with HIV, HBV or other hepatitis viruses were excluded from the study; samples with scarce RNA were also excluded. Clinically healthy individuals with normal ALT values and negative tests for HCV infection and other viral diseases were selected for the non-infected individuals (*n *= 17).

HCV-infected patients (*n *= 48) were treated with a combination of pegylated IFN-α 2a (180 μg/week) and ribavirin (1000-1200 mg/day, Roche) for 48 weeks; in accordance with the hospital management rules. Eleven infected patients were analyzed prior to treatment and were considered as untreated (U) for this study. Viral loads were monitored in all infected patients with a periodicity of 6 months. Response to IFN therapy was determined on the basis of viral load reduction or virus clearance at 6 months after treatment end.

The study was carried out in accordance with ethical regulations approved by the institutional committee and in accordance with the Declaration of Helsinki.

### RNA extraction

Blood samples were centrifuged at 1000 × g for 20 min and leukocyte bands were extracted and treated with red blood cell lysis buffer (0.15 M ammonium chloride, 10 mM KHCO_3_, 0.1 mM Na_2 _EDTA) at 4°C for 15 min and centrifuged at 480 × *g *for 4 min. This procedure was repeated three times. Remaining white cells were washed once with phosphate-buffered saline solution.

RNA was extracted from PBMCs with Trizol reagent (Invitrogen, Carlsbad, CA, USA) following conventional procedures. RNA was air dried and treated with DNAse I (Fermentas, Glen Burnie, MD, USA). RNA concentration and quality were determined by spectrophotometry and visualized by 1% agarose gel electrophoresis with ethidium bromide.

### Real-time RT-PCR for *ifnar1*

All RT-PCR were set up in 96-well optical plates using 4 ng of patients' RNA, 10 μl TaqMan Universal PCR Master Mix (Applied Biosystems, Foster City, CA, USA), and 1 μl of primers/probe set containing 900 nM of forward (5'-GCTTTGGATGGTTTAAGCTTTACATATAGC-3'), and reverse primers (5'-TCTGGTGAGAGTTTATAAATTTTATGTCTGGAAT-3'), and 300 nM probe [FAM]CTTCAGGTGTAGAAGAAAG[NFQ] was added to a final volume of 20 μl per reaction. *Hprt *was used as housekeeping gene. All samples were tested in duplicate. RT-PCR program consisted of incubation at 48°C for 30 min, and 40 cycles at 95°C for 10 min, 95°C for 15 sec, and 60°C for 1 min with the 7500 Real-Time PCR System (Applied Biosystems).

CT and amplification efficiencies for target (*ifnar1*) and housekeeping (*hprt*) genes were tested simultaneously and compared to demonstrate their equivalence. Results were expressed in ΔCT, indeed target gene CT minus housekeeping gene CT. After this, the comparative method 2^-ΔΔCT ^was used for relative quantification of *ifnar1 *transcription between study groups [30,31].

### Real-time RT-PCR for *socs1*

Patients' RNA samples were reverse-transcribed using the random primers included in the Two-Step RT-PCR Power SYBR Green Kit (Applied Biosystems). Reactions were done at 25°C for 10 min, 48°C for 30 min and 95°C for 5 min. *socs1 *cDNA was amplified using the Power SYBR Green Master Mix, 900 nM of forward (5'-CACGCACTTCCGCACATTCC-3') and reverse primers (5'-TCCAGCAGCTCGAAGAGGCA-3'), and 4 ng RNA in a final volume of 20 μl. Then, 40 cycles were carried out at 50°C for 2 min, 94°C for 10 min, 95°C for 2 min, 94°C for 1 min, 62°C for 30 sec and 72°C for 1 min. *hprt *was used as housekeeping gene. All reactions were done in duplicate. Dissociation curves were constructed at 95°C for 15 sec, 60°C for 30 sec, 72°C for 1 min, 45°C for 15 sec and 60°C for 15 sec; *socs1 *expression was quantified by means of the comparative method 2^-ΔΔCT ^as mentioned above.

### Statistical analysis

Results expressed as 2^-ΔΔCT ^were reported as mean ± standard deviation and were analyzed using one-way ANOVA test. Differences between more than two groups were assessed by Kruskal-Wallis test. Correlations between epidemiological variables were analyzed by the Spearman test using ΔCT values. *P *values < 0.05 were considered statistically significant.

## List of abbreviations

IFNAR: interferon-alpha receptor; IFN: interferon; SOCS: suppressor of cytokine signaling; RT-PCR: reverse-transcription polymerase chain reaction; PBMC: peripheral blood mononuclear cells; HPRT: hipoxanthine guanine phosphoribosyl transferase; HCC: hepatocellular carcinoma; HCV: hepatitis C virus; ALT: alanine amino transferase; CT: threshold cycle.

## Competing interests

The authors declare that they have no competing interests.

## Authors' contributions

VSM, GSL, RCRG, ARM, VVR and JRL participated in the study design, performing of molecular tests, data analysis, drafting and discussing of manuscript. DMM attended patients infected with hepatitis C virus and assessed medical treatment and laboratory studies and participated in the analysis of clinical-epidemiological data. All authors read and approved the final manuscript.
